# Laparoscopic management of infantile hydrocele in pediatric age group

**DOI:** 10.1007/s00383-022-05064-8

**Published:** 2022-02-06

**Authors:** Ahmed Elhaddad, Mohamed Awad, Sherif M. Shehata, Mohamed A. Shehata

**Affiliations:** Department of General Surgery, Pediatric Surgery Unit, Faculty of Medicine, Tanta University, Egypt, El-Geish Street, Tanta, 31527 Egypt

**Keywords:** Hydrocele, Laparoscopic hydrocelectomy, Internal inguinal ring, Laparoscope in pediatrics

## Abstract

**Purpose:**

To evaluate laparoscopic management of hydroceles in pediatrics, with evaluation of the internal inguinal ring (IIR) and the PPV (patent processus vaginalis) in different types of hydroceles, and the incidence of the contralateral PPV.

**Methods:**

The IIR and the type of hydrocele on the same side of 93 patients with 106 infantile hydroceles were evaluated and managed, in addition to contralateral side.

**Results:**

The IIR on same side was closed in 8.5% (Type I) and patent in 91.5% (Type II and III) with different shapes. Contralateral IIR was open in 88.7% of cases. The operative time was 30.99 ± 7.23 min, with no intra-operative complication. The vas deferens and testicular vessels were secured and there were no injuries or bleeding. The conversion rate was zero, and all procedures (Type II and II) were completed totally laparoscopic. No post-operative complications except a case of tense hydrocele developed scrotal edema that managed conservatively.

**Conclusion:**

Laparoscopic hydrocelectomy is safe, applicable and feasible for management of different types of hydroceles in pediatrics. The IIR is patent in nearly all cases with/out communication to the hydrocele. The contralateral IIR can be managed in the same session. Laparoscopic hydrocelectomy with/out hydrocelectomy and IIR closure is essential in preventing recurrence.

**Supplementary Information:**

The online version contains supplementary material available at 10.1007/s00383-022-05064-8.

## Introduction

Infantile hydrocele is an abnormal collection of fluid along the course of the processus vaginalis due to incomplete obliteration. The occurrence of infantile hydrocele is related to the descent of the testis, as it passes through the internal ring, it pulls along a diverticulum of peritoneum on its anteromedial surface referred to as “the processus vaginalis” [1].

Persistent patent processus vaginalis (PPV) is a common cause of hydrocele in children and explains approximately 60% of the cases in infants. So, closure of the PPV may be the most effective in preventing the recurrence [2].

Traditional open repair entails performing an inguinal incision, dissecting the inguinal canal, high ligation of the PPV, and draining the fluid or window created in the tunica vaginalis [3]. However, Laparoscopic closure of the internal orifice of the PPV became an option for the treatment of hydroceles in children [4].

The timing of surgical intervention was one of the following conditions according to the survey of the Section on Surgery of the American Academy of Pediatrics: appearance of hydrocele after one year of age, initial onset in infancy but persistence beyond one year of age, and presence of a reducible or communicating hydrocele [3, 5–7].

The aim of this study was to evaluate the applicability, efficacy and safety of laparoscopic management of hydrocele in the pediatric age group aiming for uniform national guidelines in the indicated children for surgery, in addition to the laparoscopic evaluation of the internal inguinal ring and PPV in different types of pediatric hydroceles, this was the primary outcome. The secondary outcome was evaluation of the incidence of the contralateral PPV.

## Materials and methods

This prospective study was conducted on 93 male children with 106 hydroceles, in the period from July 2019 to June 2021, at the pediatric surgery unit, surgical department, Tanta university hospital and its affiliated hospitals, Tanta, Egypt. After approval from the institute’s Research Ethics committee, an informed consent was taken from parents or the legal guardians of each patient. The privacy of participants and confidentially of the data were considered and patient ID for each participant.

**Fig. 1:**
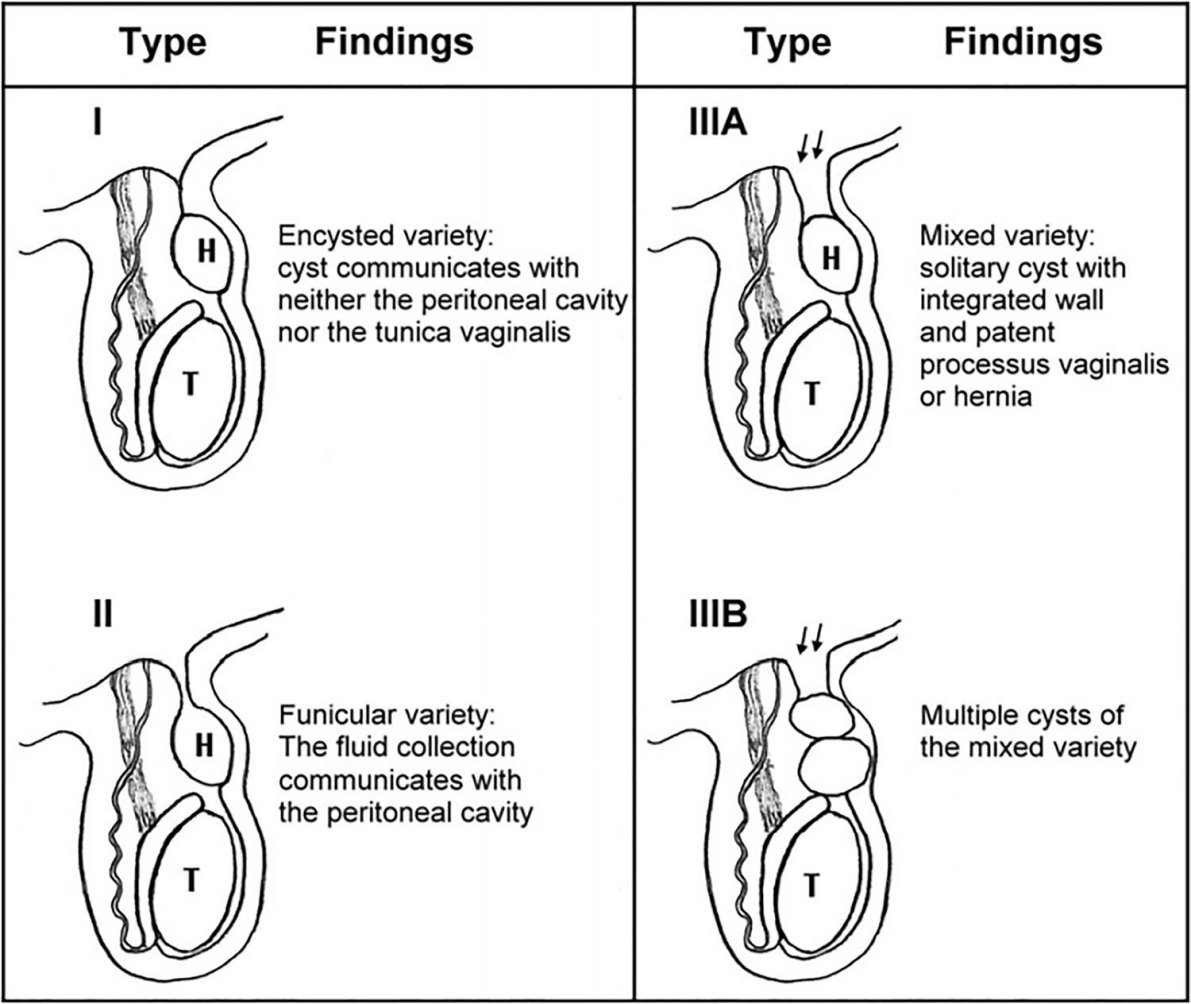
Schematic diagram showing classification of hydrocele according to the relationship between the cord hydrocele and the processus vaginalis. Arrows: patent processus vaginalis. *H* spermatic cord hydrocele, *T* testis “Chang et al. [2]”

We included in this study patients presented with hydrocele after one year of age, patients with initial onset of hydrocele in infancy but persistent beyond 1 year of age, presence of a reducible or communicating hydrocele. We excluded cases of Type I hydroceles described by Chang et al. (Fig. 1) as they had closed IIR (the cyst does not communicate with the peritoneal cavity) [2].

Preoperatively, all patients underwent thorough clinical examination and evaluation of the inguinoscrotal region, inguinoscrotal ultrasonography, routine pre-operative laboratory investigations were done, parents of the patients were informed about the advantages and disadvantages of the laparoscopic surgery and signed a consent for the surgery.

Under general anesthesia, in the supine position, the operator and camera man stand at the head of the patient and the monitor at the end of the operating table. Longitudinal trans-umbilical incision was performed and 5-mm trocar for the scope was inserted and secured to the abdominal wall, pneumo-peritoneum created followed by exploration of the abdominal cavity and internal inguinal ring (IIR) on both sides, afterwards another two working trocars (3-mm or 5-mm) were inserted under vision on the right and left midclavicular line at the level of the umbilicus.

The shape of the IIR on the same side was evaluated laparoscopically and classified according to the type of the hydrocele described by Chang et al. (Fig. 1) [2], into Type I with *closed IIR* and no communication between the hydrocele and peritoneal cavity (excluded from studied cases due to closed IIR), Type II *opened IIR* with communication between the hydrocele and peritoneal cavity, Type III the IIR is *wide open* and the hydrocele does not connect to the peritoneal cavity.

For Type II communicating hydrocele, the IIR was dissected like that of inguinal hernia followed by complete excision of the hydrocele or going as far as possible beyond the narrow part to avoid recurrence in the remaining part of the sac, the conjoint tendon was sutured to the ilio-pubic tract and the peritoneum was closed (Video 1).

The laparoscopic management for type III hydrocele either A or B involved dissection of the IIR and delivery of the encysted hydrocele with either single or double cysts followed by wide elliptical excision of the wall and then closure of the muscle arch and peritoneum.

The contralateral IIR was evaluated either it was closed or open, if it was open, dissection of the IIR followed by excision of the sac as far as possible and closure of the peritoneum with or without muscular arch repair.

Unless there were any post-operative complications, the patients were discharged home on the same day, and follow-up every week during the first month then after 3, 6, and 12 months. All our cases were followed up clinically in regular visits in outpatient clinic and post-operative ultrasound was not routine for post-operative follow-up, just indicated in case of presence of post-operative recurrence of hydrocele that detected clinically.

## Results

**Fig. 2:**
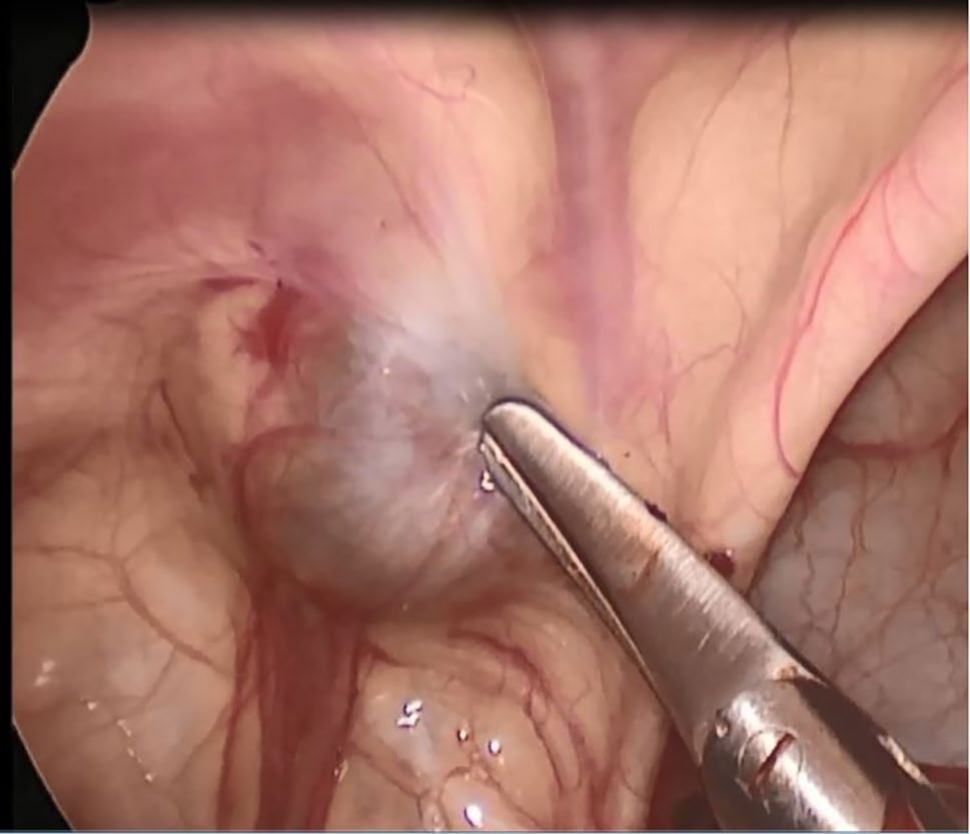
Type I; closed IIR and no communication to the peritoneum

This study included 93 male patients with 106 hydroceles, bilateral hydroceles detected by clinical examination in 13 patients (14%), right side hydroceles were in 49 patients (52.7%), and left side were in 31 patients (33.3%). After exclusion of 9 cases (8.5%) (Type I) in which the IIR was closed and all were unilateral (Fig. 2), of the remaining 71 patients with unilateral hydroceles, patent contralateral internal ring was detected by ultrasound examination in 9 patients (12.7%). During laparoscope, patent contralateral internal ring was detected in 54 of the remaining 62 patients (87.1%). The age of the participants ranged from 1 to 72 months old with a mean of 24.08 ± 14.73 months. The included hydroceles Type II and III were all completed laparoscopically with no conversion to open surgery during the period of the study.

**Fig. 3:**
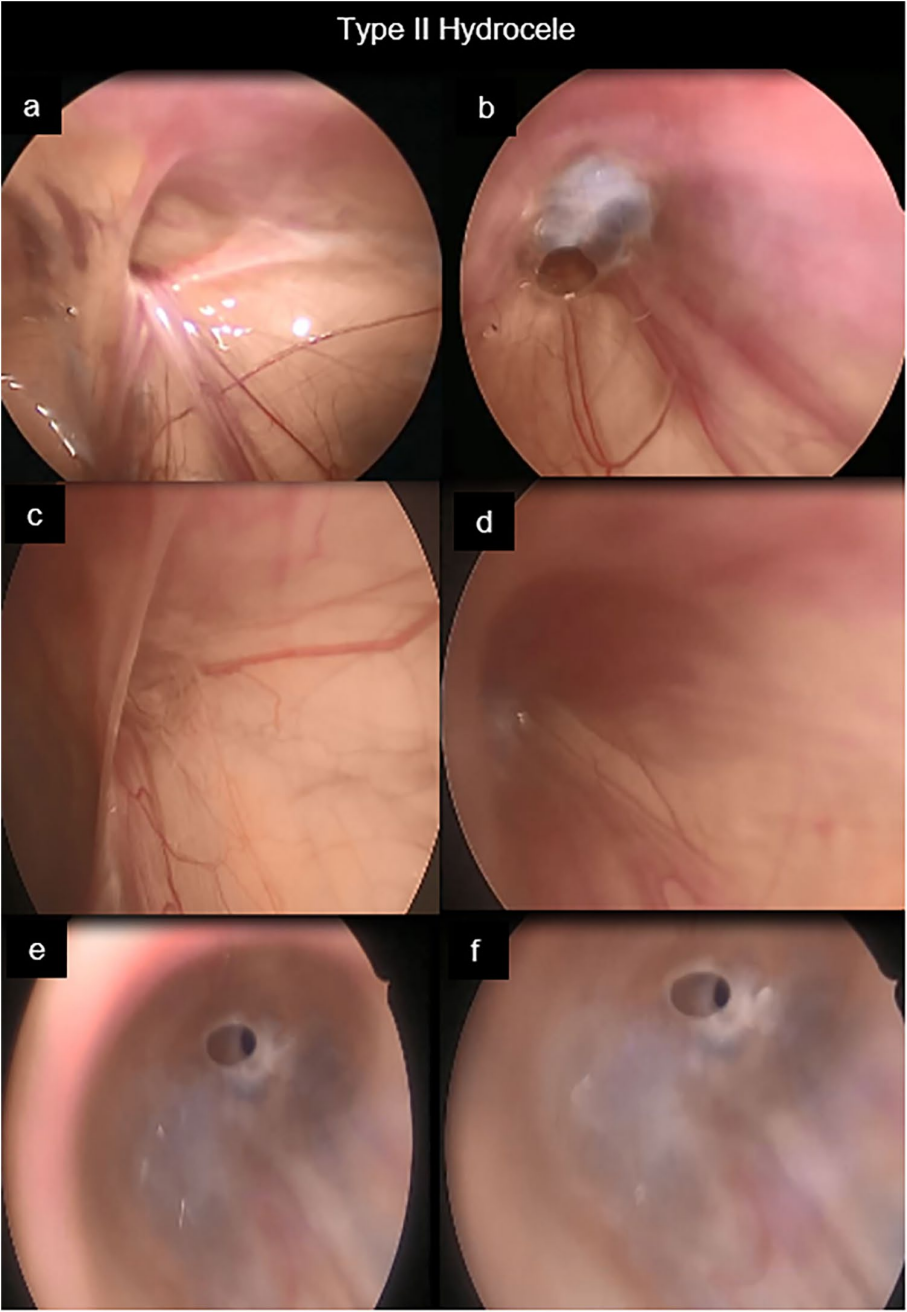
Type II **A**; **a** & **b**, **a**: the IIR is open and wide, **b**: the hydrocele connected to the ring through a valve. Type II **B**; **c** & **d**, **c**: the opening covered by peritoneal seal, **d**: IIR is open. Type **C**; **e** & **f**: open IIR with pin hole communication

**Fig. 4:**
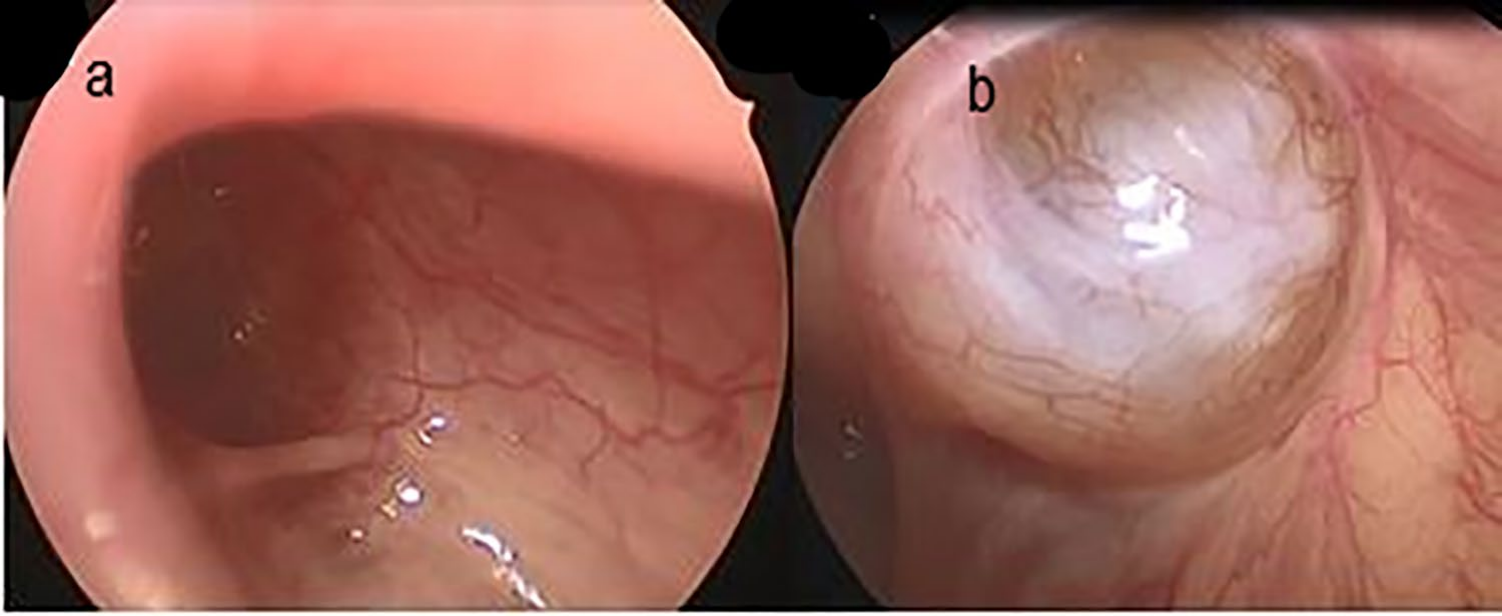
Type III; **a**: open IIR, **b**: external compression there were no communication

As regard to the laparoscopic shape of the internal inguinal ring (IIR) on the same side, we found that the IIR was patent (Type II and III) in 97 hydroceles (91.5%) (Figs. 3 and 4) (Table 1) (video 2).

**Table 1:** Shape of the internal inguinal ring by laparoscope on the hydrocele side

Total number of hydroceles	106 (100%)
**Type I** (closed ring, with **no** communication to peritoneal cavity) **(Excluded)**	9 (8.5%)
**Type II** (patent ring, hydrocele communicate to peritoneal cavity) [78 (73.6%)]	
**A** (wide opening)	35 (33%)
**B** (covered by peritoneal seal)	14 (13.2%)
**C** (pin hole)	29 (27.4%)
**Type III** (patent ring, hydrocele does **not** communicate to peritoneal cavity) [19 (17.9%)]	
**A** (solitary cyst)	16 (15.1%)
**B** (multiple cysts)	3 (2.8%)

According to these findings and classification the procedure performed as follow. Type II (Communicating hydrocele) (78 hydroceles) was managed through excision of the sac as far as possible, evacuation of hydrocele then closure of the IIR. Type III A and B were managed similarly in addition to delivery of the encysted part (one or two cysts), evacuation followed by excision of a wide ellipse of the wall.

The contralateral IIR was found to be open in 63 (88.7%) of the remaining 71 patients, dissection of the sac and closure of the IIR was done.

The operative time ranged from 20 to 45 min (for one side) with a mean of 30.99 ± 7.23 min, with no intra-operative complications. The vas deferens and testicular vessels were secured and there were no injuries or bleeding. The open conversion rate was nil, and all procedures completed totally by laparoscopy.

All patients were followed up in the outpatient clinic. The mean follow-up period was 13.8 ± 4.1 months (range from 6 to 23 months), and there was no evidence of recurrent hydrocele or testicular atrophy, post-operative ultrasound was not routine for post-operative follow up. We just did ultrasonography post-operatively in one patient with post-operative scrotal oedema that revealed no recurrence and was managed conservatively.

## Discussion

The frequency of pediatric congenital hydroceles is reported to be about 5.7% and there were many classifications describe the pathology. Martin et al. described two types of hydroceles either funicular type in which the peritoneal diverticulum communicating with the peritoneal cavity at the internal inguinal ring or the encysted type in which the cyst not communicating with the peritoneal cavity or processus vaginalis [8]. Our results was matched with the Chang et al. classification which categorized hydroceles that do not belong either funicular or encysted type as mixed type, In which the cyst is not communicating with the peritoneal cavity but has a proximally patent processus vaginalis [2].

Based on our results, we can modify the previous hydrocele classification of Chang et al. [2] which was described in (Fig. 1), where we can add subdivision to Type II. Type II A IIR was wide opening, Type II B covered by peritoneal seal, Type II C narrow communication with hydrocele (pin hole) (Fig. 3) (Video 2).

The ideal time for congenital hydrocele repair is controversial, because most of PPV will spontaneously close within 1–2 years. Therefore, most surgeons may avoid hydrocele operation within 1–2 years of life unless hernia cannot be excluded [3]. In our study we included patients with appearance of hydrocele after 1 year of age, or persistent beyond 1 year of age, and patients with reducible or communicating hydrocele. We found that the operated patients with hydrocele under 1 year of age were 5 cases and there were all communicating hydrocele (Type II A) with wide patent processus vaginalis. Other study described the operation in the first year of life only required if it is huge in size or associated with inguinal hernia [9]. In contrast, others reported that in the case of hydroceles with PPV, elective operation is recommended regardless of age since there is a high risk of hernia to develop due to PPV [2]. Choi et al. in their comparative study restricted the age after two years except if comorbid ipsilateral inguinal hernia or cryptorchidism that mandate surgery before that age [10].

Janetschek et al. in 1994 was the first to perform laparoscopic hydrocelectomy [11]. Takehara et al. began successfully using laparoscopic percutaneous extraperitoneal closure (LPEC) to treat children with inguinal hernias [12]. Since then, modified LPEC techniques have been reported, which differ from each other in the use of LPEC surgical devices, including self-made hernia needles, Endoclose needles, GraNee needles, Reverdin needles, subcutaneous injection needles, common suture needles and epidural needles as suturing instruments [4, 13]. The recurrence rate was higher with the use of percutaneous techniques described by Zahng et al. [4] the cause of recurrence was due to reopened or mis-ligated PPV in the open group or ligature loosening that resulted in incomplete closure of the PPV in laparoscopic group. Also, Shehata MA study did not recommend LPEC due to high rate of complications and recurrence [14]. In our study all procedures were performed totally laparoscopic using three ports.

Many surgeons stated that laparoscopic surgery is only indicated for communicating hydroceles. However, Yang et al. in a 10-year experience and follow-up of laparoscopic repair of hydroceles of all types reported that 283/284 patients (99.6%) in their case series were discovered with open internal rings and PPV instead of closed internal rings whatever the type of hydrocele [15].

Moreover, Zhang et al. reported that open PPV was found at the internal ring orifice in 98.53% of patients during laparoscopic surgery and ideal efficacy was achieved following the closure of the internal ring and percutaneous aspiration through the scrotum. Furthermore, 1.47% were confirmed to have a negative PPV or internal ring orifice and these patients were switched to the trans-scrotal procedure resulted in minimized surgical incisions compared with conventional inguinal approach [4].

The laparoscopic approach has the advantages of less injury to the spermatic cord and spermatic duct, more cosmetic incisions and the possibility of finding and treating contralateral PPV and other abnormalities [2, 10].

In comparison for the results of Choi et al. who described patent IIR in all cases [10], we found that the IIR was closed in 9 (8.5%) hydroceles and patent in 97 (91.5%) hydroceles. However, this study matched with the results of Saka et al. who reported 97.7% of hydroceles were patent around the internal inguinal ring: 59.1% narrow patent processus vaginalis covered with peritoneal veil, and 38.6% widely open patent processus vaginalis [16].

Our results (9 cases Type I with closed IIR) matched with Zahng et al. who had fourteen cases with closed IIR and were converted to open scrotal approach for repair [4].

The recurrence rate after laparoscopic hydrocelectomy was reported to be 0–1.4% [15, 17, 18]. In our study, there was no recurrence of hydrocele after total laparoscopic hydrocelectomy for all types. However, in Choi et al. study, there was one (0.7%) recurrence in scrotal incision hydrocelectomy (SIH) group and the recurrence rate of the whole study was 0.2% in a comparative study with SIH and total laparoscopic hydrocelectomy (TLH) [10].

When evaluation of the contralateral IIR, our approach of total laparoscopic three ports technique allowed good visualization and detected that 88.7% of cases had contralateral PPV in comparison to Zahng et al. study of different LPEC who reported that the two-port LPEC approach is better for diagnosing contralateral PPV and reducing metachronous hernia or hydrocele than the single-port LPEC procedure [4].

There is a controversy about operating on the contralateral side of hydrocele especially when it is not clinically relevant, but in the study, we operated on the contralateral side as well to solve the hydrocele and to safe the patient another operation in the future especially when there was patent processus vaginalis or communicating hydrocele.

In our study all contralateral PPV was managed laparoscopically with complete dissection of the ring, excision of the sac and IIR closure. However, in the literature, the treatment of contralateral PPV remains controversial and the probability of hernia or hydrocele if left untreated is approximately 5.6–16% [19]. Zahng et al. recommended that all types of contralateral PPV should be treated, and advised ligation if the opening larger than 2 mm, and the peritoneal orifice is torn with forceps when its diameter less than 2 mm [4].

## Conclusion

Laparoscopic hydrocelectomy is safe, applicable and feasible for management of different types of hydroceles in pediatric age group. The IIR is patent in nearly all cases with or without communication to the hydrocele. Laparoscopic hydrocelectomy with IIR closure is essential in preventing recurrence. The contralateral IIR can be managed laparoscopically in the same session.

## Limitations

This study had some limitations, small number of patients for common surgical entity. It was relatively a short-term prospective study at a single tertiary center. Total laparoscopic hydrocelectomy was performed for all types with no comparison to the laparoscopic-assisted or the conventional open procedures and further studies for comparison are recommended.

## Supplementary Information

Below is the link to the electronic supplementary material.

**Video 1:** Type II hydrocelectomy (MP4 103540 kb)

**Video 2:** Types of hydroceles (MP4 118328 kb)

## Data Availability

The datasets used and/or analyzed during the current study are available from the corresponding author on reasonable request.

## Abbreviations

IIR: Internal inguinal ring
LPEC: Laparoscopic percutaneous extraperitoneal closure
PPV: Patent processus vaginalis
SIH: Scrotal incision hydrocelectomy
TLH: Total laparoscopic hydrocelectomy

## Declarations

### The committee’s reference number

33296/08/19.
